# Using qualitative research and the person-based approach to coproduce an inclusive intervention for postpartum blood pressure self-management

**DOI:** 10.1136/bmjopen-2024-098162

**Published:** 2025-06-24

**Authors:** Cynthia Akelo Ochieng, Carol Burke, Marcus Green, Sandra Igwe, Katherine Louise Tucker, Lisa Hinton, Lucy Goddard, Cristian Roman, Richard J McManus, Lucy Yardley

**Affiliations:** 1 School of Psychological Science, University of Bristol, Bristol, UK; 2 Action On Pre-Eclampsia, Evesham, UK; 3 Nuffield Department of Primary Care Health Sciences, University of Oxford, Oxford, UK; 4 The Motherhood Group, London, UK; 5 Institute of Biomedical Engineering, Department of Engineering Science, University of Oxford, Oxford, UK; 6 Brighton and Sussex Medical School, Universities of Sussex and Brighton, Brighton, UK; 7 School of Psychology, University of Southampton, Southampton, UK

**Keywords:** Self-Management, Postpartum Women, Patient Participation, Telemedicine, Behavior, QUALITATIVE RESEARCH

## Abstract

**Abstract:**

**Objective:**

To coproduce an inclusive intervention for blood pressure (BP) self-management post partum.

**Design:**

Using the person-based approach, an intervention was coproduced in three phases. Phase 1 entailed intervention coproduction with a diverse patient and public involvement panel and stakeholders (clinical, academic, government and third sector-based). Phase 2 involved intervention optimisation through think-aloud interviews with former patients and clinicians. Phase 3 was user-testing followed by semistructured interviews with current patients and their clinicians.

**Setting:**

Patients and clinicians from primary and secondary care drawn from Southern and Northern England.

**Participants:**

Seven former patients and 11 clinicians participated in think-aloud interviews to provide their views of intervention prototypes (phase 2). Additionally, 23 patients and 9 of their clinicians participated in semistructured interviews after using the intervention for 2 weeks (phase 3).

**Intervention:**

An interactive patient app—My BP Care—and accompanying leaflet to support BP self-monitoring. These were linked to a clinician dashboard with alerts and an emailing system to facilitate appropriate titration of patient medication.

**Results:**

The intervention was codeveloped following these guiding principles to ensure it was accessible and inclusive: easily comprehensible, motivating, simple and quick to use. Interview findings indicated that patient adherence to the intervention was promoted by the initial patient training conducted by the midwives, the enhanced clinical oversight they felt they received as a result of the intervention, the free BP monitor they received, reassurance they received of the medication safety for them and their baby, the intervention’s simplicity and the motivating reminders they received.

**Conclusions:**

Through coproduction with a diverse group of patients and stakeholders, and optimisation through testing among further diverse patients and clinicians, we developed a multicomponent intervention that is accessible and engaging for diverse patients, compatible with prevailing clinical practice and adaptable to different clinical contexts.

**STRENGTHS AND LIMITATIONS OF THIS STUDY**

- A major strength of this study is its iterative recruitment and involvement of a diverse group of patients including those disproportionately affected by postpartum hypertension, clinicians and other key stakeholders.
- Data collection included real-life user-testing and optimisation with current and former patients and clinicians and proceeded until data saturation was achieved.
- Data analysis was conducted iteratively and involved regular team meetings to discuss the findings.
- A limitation of this study is that there was no recruitment of clinicians working in primary care for user-testing.

## Background

 Hypertensive disorders of pregnancy (HDPs) are increasingly prevalent (between 7% and 15%) and are associated with maternal and perinatal adverse outcomes.^1^ HDPs include chronic hypertension, gestational hypertension and pre-eclampsia/eclampsia.^2^ HDPs can lead to both fetal and maternal complications in the short and long term—including cardiovascular disease, stroke, renal complications and even death.^2^ Better management of HDPs post partum is associated with reduced risk of developing chronic hypertension and longer-term cardiovascular disease.^3^ While evidence exists for the importance of managing blood pressure (BP) during pregnancy, more research is required on its management post partum. Historically, it was thought that once the placenta was removed, HDPs like pre-eclampsia and gestational hypertension would be resolved naturally by the body.^4^ However, it is now also known that BP can change very rapidly post partum,^5^ patients, therefore, need to be monitored closely to ensure that they get appropriate and timely treatment.^3^

In the UK, the guidance is for patients to be monitored up to every other day.^6^ This level of close monitoring of patients post partum is, however, an ideal that is often a challenge within many health systems including the UK National Health Service (NHS).^7^ One way to enhance the feasibility of this close monitoring is through patient self-management. In previous studies (Self­maNAgement of Postnatal anti­Hypertensive Treatment (SNAP-HT) and Physician Optimized Postpartum Hypertension Treatment Trial (POP-HT)), following self-management through a digital intervention for self-recording of BP, remote clinical oversight and subsequent medication adjustments, patients had better controlled BP at 6 months post partum and up to 4 years in longer-term follow-up.^8^,^10^ However, in both studies, the patients were primarily white and middle class. Findings from both studies recommend that further research be conducted with more diverse patient groups.^8 10^ This is particularly important given that prevailing disparities—maternal mortality is four times higher in black women than in white ones, 60% of deaths following pregnancy were associated with pre-existing conditions such as hypertension and ethnic minorities are over-represented among those deaths. Multiple disadvantages—such as living in a more deprived area, being on lower income or lower education status—further exacerbate worse outcomes.^11^ Both POP-HT^10^ and SNAP-HT^8^ focused on patients with gestational hypertension. There was a need to develop an intervention that was suitable for patients from different ethnic backgrounds, living in more and less deprived areas, with different education levels and with HDPs including gestational and chronic hypertension. The intervention needed to be usable not just in different NHS Trusts, primary and secondary care facilities but also by a diverse group of patients in different contexts.

Unfortunately, in many countries including the UK, the service provision in the puerperium (first 6 weeks after the pregnancy) is often varied with some patients lacking adequate clinical follow-up.^7^ Among patients for whom clinical care is inadequate, there can be a lack of clarity on whether to continue taking medication, which medication and how much to take and which clinician to contact to help manage their BP; this results in gaps in clinical care and increased morbidity and mortality.^12^ This is further exacerbated among underserved populations. For example, previous research in high-income countries found worse postpartum BP outcomes among black women.^13^,^15^ Baiden *et al*^16^ explain that this increased risk and associated negative outcomes are due to the intersectionality of disadvantage among these populations that compounds the inequity in health.

A key aim of this study was to develop an inclusive intervention for BP management post partum that could be used among underserved communities, thereby promoting health equity. The intervention also needed to be feasible and pragmatic enough to be adopted into usual care in different clinical contexts within the UK. The success of the intervention required input from both the patients and the supporting clinicians. In order to promote both patients’ and clinicians’ engagement with the intervention, it needed to be as acceptable, accessible and motivating as possible.^17^ The person-based approach (PBA) was applied to ensure that the intervention designed was rigorously grounded in the psychosocial and organisational context of the intervention users.^18^ Through PBA, interventions are designed to promote their appropriateness, feasibility and effectiveness while also aligning with behaviour change theory.^17^ Previous evidence-based interventions were used as a basis for the development of this intervention that would suit the diverse needs of the different patients and clinical contexts.^89 19^,^21^

## Methods

The methods and findings described here follow the GUIDance for the rEporting of intervention Development framework^22^ and the Template for Intervention Description and Replication guidance^23^ for reporting intervention development studies. The Medical Research Council (MRC) framework for complex interventions was applied in this study. It assists with prioritisation of research questions, design and methods and encourages the development and use of more promising approaches.^24^ The PBA which is applied in this study was developed from the MRC guidance^25^ as one of the promising approaches and is recognised within the MRC guidance as such.^26^

The main purpose of this study was to develop an inclusive intervention for BP management post partum that could be used among underserved communities and in different contexts. Success of the intervention will result in better BP management post partum which is associated with reduced risk of developing hypertension and cardiovascular disease. Intervention development proceeded in three main phases: initial coproduction of the prototype elements with patient and public involvement (PPI) contributors and stakeholders (phase 1); optimisation with former patients and current clinicians through think-aloud interviews (phase 2); and finally, optimisation through qualitative feedback based on real-world user-testing by current patients and their clinicians within NHS Trusts (phase 3). Each phase of development is explained below, in the order in which they were conducted. PPI was involved in the phase 1 coproduction and then contributed and provided valuable feedback throughout phase 2 and phase 3 optimisation; every change to the patient-facing elements and patient pathways was discussed with PPI before being adopted.

### Phase 1

Phase 1 of the study was intervention planning and codesign of the first prototype. This involved collating evidence from previous studies on BP self-management post partum and discussion with PPI and other stakeholders (clinicians, researchers, senior health-system leaders, senior government advisers and third sector-based).

#### Patient and public involvement

PPI was involved in the phase 1 coproduction and then contributed and provided valuable feedback throughout phase 2 and phase 3 optimisation; every change to the patient-facing elements and patient pathways was discussed with PPI before being adopted.

#### PPI and stakeholder recruitment

The PPI group comprised 14 individuals of varied ethnic backgrounds (black, Asian and white). Most lived in high-deprivation areas, with different employment statuses, religious affiliations and educational levels. The recruitment process remained flexible to enhance diversity. 15 stakeholders were recruited via referrals from the study team throughout the study. The group was multidisciplinary, each with an interest in maternal health and equity.

#### PPI and stakeholder coproduction

PPI and stakeholders participated in regular meetings to inform the intervention development. PPI discussed their experiences and ideas for the intervention, while stakeholders provided feedback on clinical and pragmatic aspects. PPI were paid £25 per hour for their involvement in line with the National Institute for Health and Care Research(NIHR) recommendations.^27^

This input, along with evidence from previous studies and behaviour change theory, was used to create logic models (online supplemental file 1) and an intervention planning table outlining the target behaviours, their facilitators and barriers, and the intervention ingredients needed to address them (online supplemental file 2). The intervention planning table collates different types of evidence on what is required within the intervention and why.^28^ In line with PBA, guiding principles (see table 1) required to make the intervention acceptable, feasible and engaging^28^ were also developed from the literature, PPI and stakeholder input. These were based on user context, design objectives and key intervention features. The process aligned with social cognitive theory, focusing on cognitive (knowledge, expectation and attitude), behavioural (skills, practice and self-efficacy) and environmental factors (access, influence on others and social norms).^29^

**Table 1:** Guiding principles

User context	Key design objective	Key intervention features
Patients often not involved in their BP management relying instead on monitoring from very busy clinicians	Provide patients with skills for self-monitoring BP	Train patients on self-monitoring their BP before discharge from hospital
	Enhance patients’ self-efficacy for BP self-monitoring	Provide patients with motivating information on the importance, benefits and safety of self-monitoring and timely medication changes in the puerperium Provide the patients with additional leaflets/booklets with motivational messages on importance and benefits of self-monitoring Highlight that by self-monitoring they will be helping and working with their clinician to help them in the intervention Highlight that the medication changes have been prepared by their clinician and that the clinician will be able to see their record from the intervention
Patients are often too busy with the newborn to prioritise their own BP management	Make the intervention simple and quick to use	Design the intervention with a few pages and simple instructions
	Make the intervention feasible in the busy lives of a postpartum patient	Design the intervention to be able to remind patients to self-monitor
Patients sometimes do not take their medication as required	Make the medication changes easy to understand	Have the medication record in the intervention written in clear simple language
	Ensure the patients have access to the required medication	Preplan that the patients are discharged with 2 weeks worth of the medication required Ensure the patient’s GP receives a letter detailing the patient’s enrolment in the study and their medication as well as how to access the self-monitoring record Include an option for patient’s clinician to change the medication in the intervention if required
BP management inefficiencies amplified in underserved communities	Make the intervention easy to understand and use by different patients	Use simple language and lots of self-explanatory graphics Ensure the intervention is compatible with other tools used to aid understanding for example, reading out loud tools, magnification apps and translation tools Develop the intervention with PPI input from different backgrounds including less heard populations groups
	Ensure equitable access to self-monitoring resources	Provide patients with the resources to self-monitor like a BP monitor, access to a smart phone and/or top-up, making the intervention free to download and use, as well as aiding them to download the intervention
Clinicians being concerned about patient safety when adopting interventions	Motivate clinicians about the safety, benefits and efficacy of the intervention	Use credible evidence to educate clinicians on the benefits and safety in patient self-monitoring Provide clinicians information highlighting how the intervention will save clinicians time and effort while assisting patients in a more timely manner
Clinicians not wanting unnecessary additional work	Make the intervention compatible with usual care while reducing time spent accessing patient BP record, making the decision for medication changes easier	Design the intervention with a page accessible to clinicians showing BP history alongside medication taken over time Design additional clinician reference material, for example, collating key NICE guidance on management of BP post partum including how to adjust medication in response to patient BP trends

BP, blood pressure; GP, general practitioner; NICE, National Institute for Health and Care Excellence; PPI, patient and public involvement.

As part of the intervention codesign, ideas were sought from previous intervention development studies for BP^810 19^,^21 30 31^ and presented to PPI. Through PPI and stakeholder meetings, draft pages of a digital intervention were cocreated in Figma (www.figma.com). After several iterations, the intervention was optimised via think-aloud interviews described in phase 2 below.

### Phase 2

In the second phase, the draft intervention components were optimised using think-aloud interviews.^32^ Think-aloud interviews involve verbalisation of thoughts while undertaking a task.^33^ Think-alouds are useful for collecting data on how users interact with an intervention and problems they may have with it, including confusing elements.^25^

#### Think-aloud interviews with past patients

Participants with HDP experience in England were recruited from diverse backgrounds through community groups in under-resourced areas of Southwest England. Interested participants received information sheets and consent forms electronically. Interviews were scheduled at the participant’s preferred location. Participants viewed the draft intervention on Figma and its accompanying leaflet while verbalising their thoughts. Probing questions were asked after content review (see online supplemental file 6 for the interview schedule). Sessions were audio-recorded with permission. Participants received a £20 voucher for their time. Their comments were compiled in a table of changes (online supplemental file 3).

#### Think-aloud interviews with clinicians

11 clinicians (midwives, obstetricians and general practitioners, GPs) were recruited through snowball sampling^34^ through the research team. They were shown templates of clinician emails that would be sent to the patients’ doctors informing them of the patient’s BP recorded through the intervention and any BP medication changes they may need to execute (see online supplemental file 7 for the interview schedule). They commented on the clarity, length, suggested actions and patient information included. Additionally, three clinicians gave feedback on the clinician medication advice document. All feedback was compiled into a table of changes (online supplemental file 3).

### Phase 3

#### PPI optimisation of the intervention

Following the think-aloud sessions, changes were made to the intervention resulting in the first prototype of My BP Care app, the leaflet, a clinician dashboard and the clinician advice document. Once the intervention was launched for mobile use, PPI tested it over a week, inputting their BP daily and receiving intervention feedback and reminders. After the week, they met with CAO to discuss their experiences, offering insights on page clarity, flow, tone and the pragmatics of the advice provided. These were all noted in the table of changes (online supplemental file 3) and actioned accordingly to further optimise the intervention.

#### Semistructured interviews with patients and their clinicians

Once the digital intervention, leaflet, clinician messages and clinician advice document had been coproduced and optimised through the think-alouds, they underwent user-testing with patients and clinicians as explained below.

##### Recruitment

Principal investigators from UK NHS maternity hospitals were invited to participate in intervention testing. Two hospitals confirmed involvement. Research midwives approached eligible patients (≥18 years, posthypertensive pregnancy) for consent. They were asked to begin using the intervention on discharge from hospital. Healthcare professionals involved were invited to participate in interviews or focus groups.

##### Protocol

At the hospital, all recruited patients were set up on the My BP Care app, provided with a free calibrated BP monitor and trained how to use both. There was the option of procuring smartphones for those who did not have a smart phone; however, all patients recruited ended up having a smart phone. Following discharge, patients checked and recorded their BP daily, receiving tailored feedback. Higher and lower values (in reference to the UK National Institute for Health and Care Excellence (NICE) guidance^35^) prompted them to contact their doctor within appropriate timelines. Simultaneously, the patient’s doctor would receive a message and flag via the intervention dashboard alerting them of the higher or lower readings requiring a medication review. When they forgot to input their BP, they would receive reminders with motivating messages. After 2 weeks, CAO conducted remote semistructured interviews (15–35 min) to gather their impressions and experiences of the intervention, challenges they were facing and how the research team could support their adherence and improve the intervention (see online supplemental file 9 for the interview topic guide). Patients received £20 vouchers for participation. Clinicians monitored the dashboard, managed high/low BP alerts and adjusted medications. CAO later conducted interviews/focus groups with clinicians to assess training, recruitment, patient adherence and intervention management (see online supplemental file 8 for the interview topic guide). These were further used to optimise the intervention.

### Data analysis

Interviews (phases 2 and 3) were conducted until data saturation^36^ was achieved—which in this case was when no new impactful changes were being suggested. The data collected were transcribed verbatim via Teams. It was then checked, corrected and anonymised. The data were organised and recorded in the table of changes (online supplemental file 3). In the table, data were categorised into positive, negative and neutral feedback for different elements of the intervention. Possible changes and reasons for change (important for behaviour change, easy and uncontroversial, mentioned repeatedly, based on experience and non-contradictory to the programme theory and evidence) for each feedback were determined. Through discussion with the research team, each possible change was prioritised based on the MoSCoW (Must have, Should have, Could have, Would Like to have) criteria.^37^ The MoSCoW criteria are an established analytical approach applied within PBA that ensures that key changes are made that are likely to impact on behaviour change and enhance an intervention’s acceptability, feasibility, persuasiveness, motivation and engagement.^30^ The intervention was optimised through this iterative analysis as documented in the table of changes (online supplemental file 3).

## Results

### Phase 1: coproducing the intervention with PPI and stakeholders

Through a combination of PPI and stakeholder input, the literature and behaviour change theory (social cognitive theory), two separate logic models (online supplemental file 1) were developed targeting patient and clinician behaviour change. The patient’s model identified four main problems: inefficient BP monitoring and medication changes, unmonitored discontinuation of medication and inequitable BP management across different population groups.

In line with Bandura’s social cognitive theory,^29^ the proposed intervention needed to address the patients’ personal (including cognitive) factors, behavioural factors and environmental (including social) factors. For example, the intervention needed to impact the patient’s knowledge, outcome expectation and attitude towards BP management. The intervention also needed to promote the patients’ skills in BP self-management and crucially promote their self-efficacy for BP self-management. The intervention also needed to incorporate environmental factors by promoting access to BP self-monitoring resources and provide a socially supportive environment through the clinical support for self-management. The intervention achieved these through containing credible information on the benefits of self-monitoring and efficient medication adjustments, training and ongoing support for self-management, targeted support and increased access among underserved populations and management of risks and expectations of the self-monitoring. Consistent with social cognitive theory, the mediating processes for this intervention involved cognitive factors such as increased belief in the efficacy and self-efficacy of self-monitoring in the postpartum period, increased positive outcome expectancies of self-monitoring and optimal BP-responsive medication changes, increased BP knowledge, increased negative outcome expectancies of poorly managed BP and reduced concerns of medication side effects. Altering these cognitive factors impacted uptake and adherence to the intervention. The clinician logic model further highlighted their need to enrol patients onto the intervention, engage with and respond to the intervention prompts, provide ongoing support to all enrolled and targeted support to underserved communities. The intervention needed to be evidence-based on the benefits and safety of self-monitoring, align with current practice, be adaptable to different clinical contexts as well as have the capacity to be delivered equitably. As with the patient logic model, the clinician logic model also aligned with social cognitive theory and represented the importance of targeting cognitive factors (outcome expectancies, attitudes and beliefs of BP self-monitoring through the intervention), behavioural factors (skills in supporting the intervention) and environmental factors (access to the intervention resources and social norms within their individual clinical context).

Following the logic model, an intervention planning table (online supplemental file 2) was cocreated with stakeholders and from input from PPI and the literature. The table listed each target behaviour and enumerated its barriers and facilitators, citing the source of information (eg, literature, PPI, stakeholder). It also enlisted the component of the intervention that would promote the facilitators and overcome the barriers and how it would do so. Some of the barriers identified were as follows: the perceived lack of time by mothers of newborns, mothers feeling too tired to self-manage their BP, forgetting to take their BP and medication, not feeling confident to take their own BP and engage with the intervention, feeling fine and not thinking that they need to take their BP or medication, needing reassurance of clinical oversight and medication safety. Barriers identified from the clinician perspective were: lacking capacity to support patients with the intervention, a lack of continuity of care into primary healthcare, uncertainty about the safety of self-management of BP post partum. Facilitators were as follows: the intervention aligning with current practice, possibility of more efficient management of patient medication titrations and a chance to empower a diverse group of patients.

Crucial to the intervention development were the guiding principles (see table 1). Through iterative discussions with PPI and the stakeholders, the principles to make the intervention pragmatic, acceptable and engaging were described. These guiding principles outlined the patient and clinician’s context, the strategic aims of the intervention design and the features of the intervention that would achieve these objectives.

### Intervention

Following the discussions above, an intervention was coproduced involving draft digital intervention (My BP Care) App pages on Figma, linked patient messages, reminders, an associated leaflet, a clinician dashboard, clinician email templates tailored to patient BP readings, and a clinician (prescription) advice document containing NICE guidance (and accessible medication adjustment tables) on the management of BP post partum. Screenshots of the patient-facing elements of the digital intervention have been included as online supplemental file 10.

The data below from phases 2 and 3 of the study were all recorded and analysed in the table of changes. Excerpts of the table of changes have been uploaded as online supplemental file 3. Additionally, the quotes illustrating the findings from the interviews reported below are detailed in online supplemental file 5.

### Phase 2: think-aloud interviews with former patients and current clinicians

Seven former patients participated in think-aloud interviews based on the intervention (My BP Care app) pages on Figma, and the leaflet. This was a diverse group of participants of different ethnicities (black, Caucasian and Asian), with different levels of education qualifications, with different religious affiliations including non-religious and different employment statuses; most lived in areas of high deprivation (see online supplemental file 4 for detailed demographic characteristics).

Having designed and optimised the intervention based on the findings represented in the logic model and intervention planning table, subsequent participant think-alouds demonstrated that by adhering to the guiding principles (see table 1), the intervention was found to be feasible, appropriate, engaging and motivating.

Participants were happy with the design, purpose and name of the intervention—My BP Care, which for them consisted of a patient app which linked to a clinician dashboard for remote monitoring, patient messages and an accompanying leaflet. They were all satisfied with its simplicity and comprehensibility of the language, navigation, contents of the pages and graphics which they found intuitive in both the app and leaflet. Participants wanted the intervention to have some clinical oversight and for that to be made explicit. They asked if they could communicate to their clinician through the app. However, this was not possible due to the extra clinical oversight and governance it would require, which would not have been compatible with nor feasible in usual care. Some participants highlighted the importance of including text assuring patients that their BP medication was safe for their breastfeeding baby. This was, therefore, included in the intervention messaging and app pages. A few participants wanted information on the side effects of the medication either included in the intervention or communicated to the patient by their clinician. It was decided among the research team that that was already covered as part of usual care. Participants also highlighted the need for reminders to patients if they forgot to submit their readings.

11 clinicians (3 midwives, 5 obstetricians and 3 GPs) participated in the think-aloud interviews for the clinician emails. They recommended summarising the email templates intended for the patients’ clinicians and to only send emails for readings that needed a medical review. It was also suggested that very brief patient details (name, NHS number, date of birth and three most recent BP readings, their medication and whether they needed medication changes) should be included in the emails. The email also included a sentence explaining how BP titration changes could be made and a link to the clinician advice document containing NICE guidance on management of BP post partum.

### Phase 3: interviews following user-testing among current patients and clinicians

#### Findings from patient interviews

26 patients were recruited across three NHS sites to test the intervention over a period of 2 weeks. 23 of those used the intervention and participated in follow-up interviews. The patients were a diverse group residing either in the North West or South Central England. They had different ethnicities (black, Asian and Caucasian), different levels of education, employment status, the majority lived in areas of high deprivation and their ages were between 27 and 45 years (see a break-down of these details in online supplemental file 4). 12 had chronic hypertension while 14 had gestational hypertension (including pre-eclampsia). They submitted a total of 499 BP readings with a median of 23 (minimum 3, maximum 43) and average of 20.8 (SD 11.4). 53 medication changes were recorded, of which 30 were self-reported by the patients and the rest were reported by their clinicians. All patients recruited were anticipated to be discharged on medication; however, for some, their elevated BPs resolved quickly post partum, necessitating halting of medication.

This user-testing was important as it highlighted whether the intervention was feasible, pragmatic, appropriate and motivating—the principles used to attain these are outlined in the guiding principles above. The interview findings demonstrated that the co-produced intervention had achieved these. For example, all the patients stated that they found the intervention easy to use and its language comprehensible, even among those not fluent in English. Patients also said that they thought it was best for them to be trained and set up on the intervention while in hospital so that they had time to explore it and ask the midwife any questions they had before they went home. This enhanced their skills and self-efficacy of self-monitoring through the intervention.

Patients expressed a positive attitude towards the intervention based on the benefits they thought it gave them, such as enhanced knowledge of their condition and its management. Patients stated that they were glad that the intervention sent them instant feedback on what to do when they input their BPs. Patients also felt reassured that their clinical team would be monitoring their readings remotely. However, they also expressed the need for out-of-hours clinical oversight for the intervention. Having their BP readings on record in the intervention was useful for patients and some showed that record to their GP; they often thought this gave their clinician the information to make a decision on their treatment. Patients were also glad that through the intervention they could view and update their medication. Following patients’ feedback, adjustments were made to the text and process of patients’ updating their medication to make it easier.

Patients also acknowledged that adhering to daily BP self-management in the puerperium was challenging. They, however, found some elements of the intervention helpful for overcoming this, such as the reminders that the App would send them when they forgot. Following their feedback, more motivating reminders were included to be sent earlier and more frequently to patients. It was also agreed that the recruiting midwives would explain to the patients that the daily readings were only for the first few weeks. If their BP stabilised, they would only need to take weekly readings. Majority of the patients recruited who did not adhere to the intervention had experienced health complications associated with the delivery or their babies.

Some patients said that it would have been better if the intervention had a section for patients to communicate with their doctor. However, it was clear that there was no capacity to monitor direct messages from patients. One of the aims of the intervention design was for it to be useable in usual care. It was therefore agreed that the patients would contact their usual clinical team if they wanted to communicate with them.

#### Findings from clinician interviews

Nine clinicians (two obstetricians and seven midwives) participated in focus groups and interviews after they had used the intervention with their patients.

At one site, clinicians said that they found recruiting to and setting patient on the study easy. They also said that patients were motivated to be enrolled because of the free BP monitors that were offered to them. Similar to the patients’ responses of increased skills and self-efficacy, the clinicians also stated that the initial training with patients was important for patient comprehension of the intervention and for stimulating their initial use of it. A midwife at a different site was, however, concerned that she might not have enough time to conduct an exhaustive training with each patient particularly with her hospital having unsteady internet. To tackle this, she suggested including some screenshots of the intervention into the leaflet for patients to refer to when they got home, this was added. Additionally, the study team created a video explaining each page of the intervention for patient reference.

To promote positive outcome expectancies and facilitate adherence from patients, a midwife said she told patients that she would be monitoring their BP readings remotely through the intervention. To enhance clinical safety of the self-monitoring, an obstetrician highlighted the need to have some order on which doctor would be responsible for the intervention, for example, actioning medication changes at particular times. Following this, it was agreed that the clinician flag on the intervention would disappear once it had been actioned. The clinicians said that they often had women running out of medication once their 2-week hospital supply was depleted and that some patients just stopped taking medication then. It was suggested that the intervention could include advice for patients to contact their GP for repeat prescriptions on discharge from hospital as a reminder.

## Discussion

A multicomponent intervention to facilitate BP self-monitoring post partum was coproduced with a diverse group of PPI and stakeholders and optimised through PPI, patient and clinician testing. The intervention included patient elements and clinician elements. The patient elements constituted BP self-monitoring through the ‘My BP Care’ app with integrated patient messages, BP feedback, motivating reminders and an accompanying leaflet. The clinician components included a clinician dashboard, clinician messages tailored to patient BP levels and requiring clinical actioning, and a clinician prescription advice document collating NICE-based recommendations on BP medication management post partum. The intervention was simple, easy to understand and quick to use and enhanced self-efficacy through providing training to the patients. It also ensured that patients had all the resources they needed for self-monitoring, including a BP monitor and medication. From a clinical perspective, the intervention was compatible with current clinical practice, adaptable to different contexts and promoted and ensured patient safety. Patient adherence to the intervention was promoted by the initial training they received from the midwives, the free monitor they received, the enhanced clinical oversight that patients felt the intervention offered, reassurance of medication safety for both mother and baby, the simplicity and clarity of the intervention and the motivating reminders they received.

A major strength of this study is that the intervention was coproduced by patients, former patients and stakeholders. It is, therefore, grounded in the psychosocial context of the patients and clinicians, making it more feasible, pragmatic, motivating, appropriate and persuasive. The study also included a diverse PPI panel ensuring that the resulting intervention was accessible and appropriate for a diverse group of patients including those from underserved communities. The study also tested the intervention in large hospitals with patients from diverse backgrounds; it was hence optimised to promote its suitability within diverse patient populations including those with worse maternal outcome statistics. While previous studies were successful among a homogeneous group of patients,^8^ this study has managed to conquer this challenge by achieving adherence from a diverse group of patients. One main weakness of this study is that it was not able to recruit GPs to test the intervention. We are, therefore, not able to report on the intervention’s acceptability, uptake and use among GPs. A trial proceeding this intervention development work will conduct a process evaluation of that aspect.

The intervention developed in this study had the duality of having non-complex recruitment procedures while being a multicomponent intervention with both patient and clinician elements working in tandem. Its key feature of being easy to use for both patients and clinicians enhanced their engagement with it and will undoubtedly be advantageous if rolled out to standard care. It provided patients with an avenue to self-manage their BP and have an accessible record of their BP. By facilitating patients recording their BP and updating their medication changes, it empowered them to take up some ownership of their data and share that with their clinician, hence taking on a more active role in their own healthcare; a key component of shared decision making as promoted by NICE.^38^ The intervention also provided patients with training and information on BP management, further empowering them through health literacy.^39^ Ultimately, the intervention promoted efficiency in medication adjustments for patients who engaged with it, resulting in better BP control in the puerperium. This is anticipated to have a significant impact on cardiac health long term.^3^ This intervention, if translated into standard care, could reduce clinician burden by facilitating a more efficient and effective way to manage BP post partum. Moreover, given its short-term and long-term health benefits, its successful translation would be evidence for its incorporation into national guidance for BP management.

Interventions for the management of BP post partum are an emerging area of research with a paucity of evidence of successful interventions.^6 40^ Published interventions include close clinical monitoring of patients^40^; however, this is often unattainable due to a lack of clinical capacity. Remote interventions for monitoring patients are being developed, including using text messaging^41 42^ and other telehealth interventions.^43 44^ While these telehealth interventions are promising, they reported that they required a nurse to assess every patient’s BP daily, make medication changes and avail themselves daily to all these patients. Due to the pragmatic constraints of that model within the UK NHS, our intervention ensured that alerts were sent to the clinical team regarding only the patients needing medication changes/closer monitoring, freeing up clinical time that would have been spent on patients who do not need a change in their management. Other interventions have focused on education and other resources for lifestyle changes^40 45^ our study, however, identified that new mothers were not able or willing to make those lifestyle changes in the puerperium. This intervention has also built on previous interventions developed in the UK^38^,^10 19 20 30^ and optimised them to suit a diverse group of postpartum HDP patients being cared for in different clinical contexts.

The aim of this publication is to document the process used to coproduce an inclusive intervention with underserved communities for BP self-management. BP management post partum is often haphazard and sub-par to the national recommendations. Underserved communities including black and ethnic minorities, those with lower income, education and living in more deprived areas are disproportionately affected and experience worse outcomes following HDPs. By coproducing an intervention with these populations and their clinicians, we were able to develop an intervention that is appropriate, effective, safe and motivating—resulting in better management of BP post partum. The resultant intervention is currently being trialled on a wider scale to assess its impact on BP across the UK (ISRCTN11042045, https://www.isrctn.com/ISRCTN11042045). In the trial, evaluations will be conducted to assess patient adherence, long-term health impact (including BP management) of the intervention and its success in different contexts including within primary care, as well as its integration into differing clinical pathways.

## Supplementary material

10.1136/bmjopen-2024-098162 online supplemental file 1

10.1136/bmjopen-2024-098162 online supplemental file 2

10.1136/bmjopen-2024-098162 online supplemental file 3

10.1136/bmjopen-2024-098162 online supplemental file 4

10.1136/bmjopen-2024-098162 online supplemental file 5

10.1136/bmjopen-2024-098162 online supplemental file 6

10.1136/bmjopen-2024-098162 online supplemental file 7

10.1136/bmjopen-2024-098162 online supplemental file 8

10.1136/bmjopen-2024-098162 online supplemental file 9

10.1136/bmjopen-2024-098162 online supplemental file 10

## Data Availability

Data are available on reasonable request.
