# Supplementary figures and images for: Using qualitative research and the person-based approach to coproduce an inclusive intervention for postpartum blood pressure self-management
Source: BMJ Open. 2025 Jun 24;15(6):e098162. doi: 10.1136/bmjopen-2024-098162 (PMC12198848; doi:10.1136/bmjopen-2024-098162)

***Supplementary file 1- logic models***


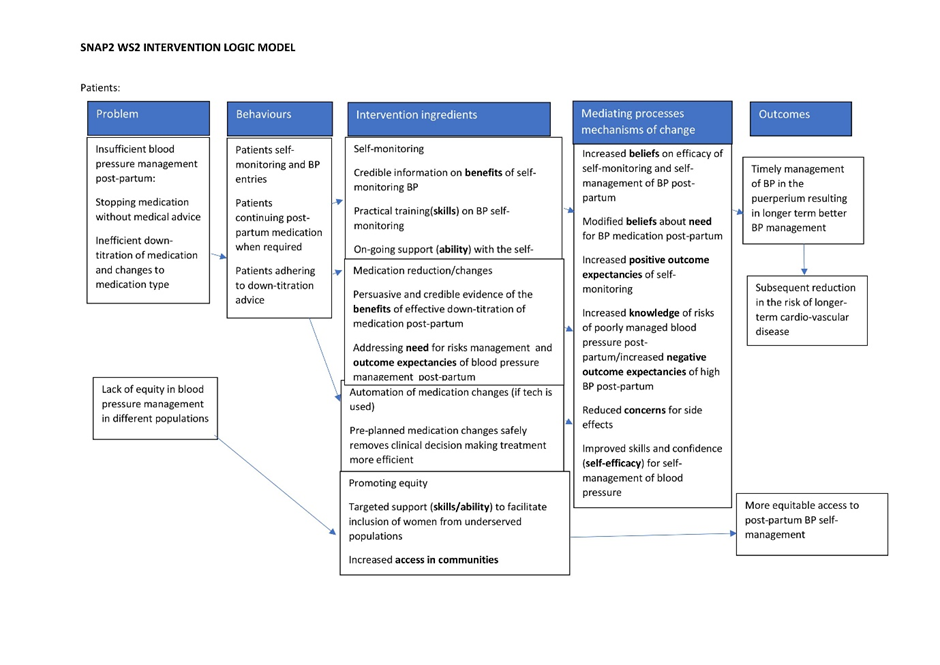


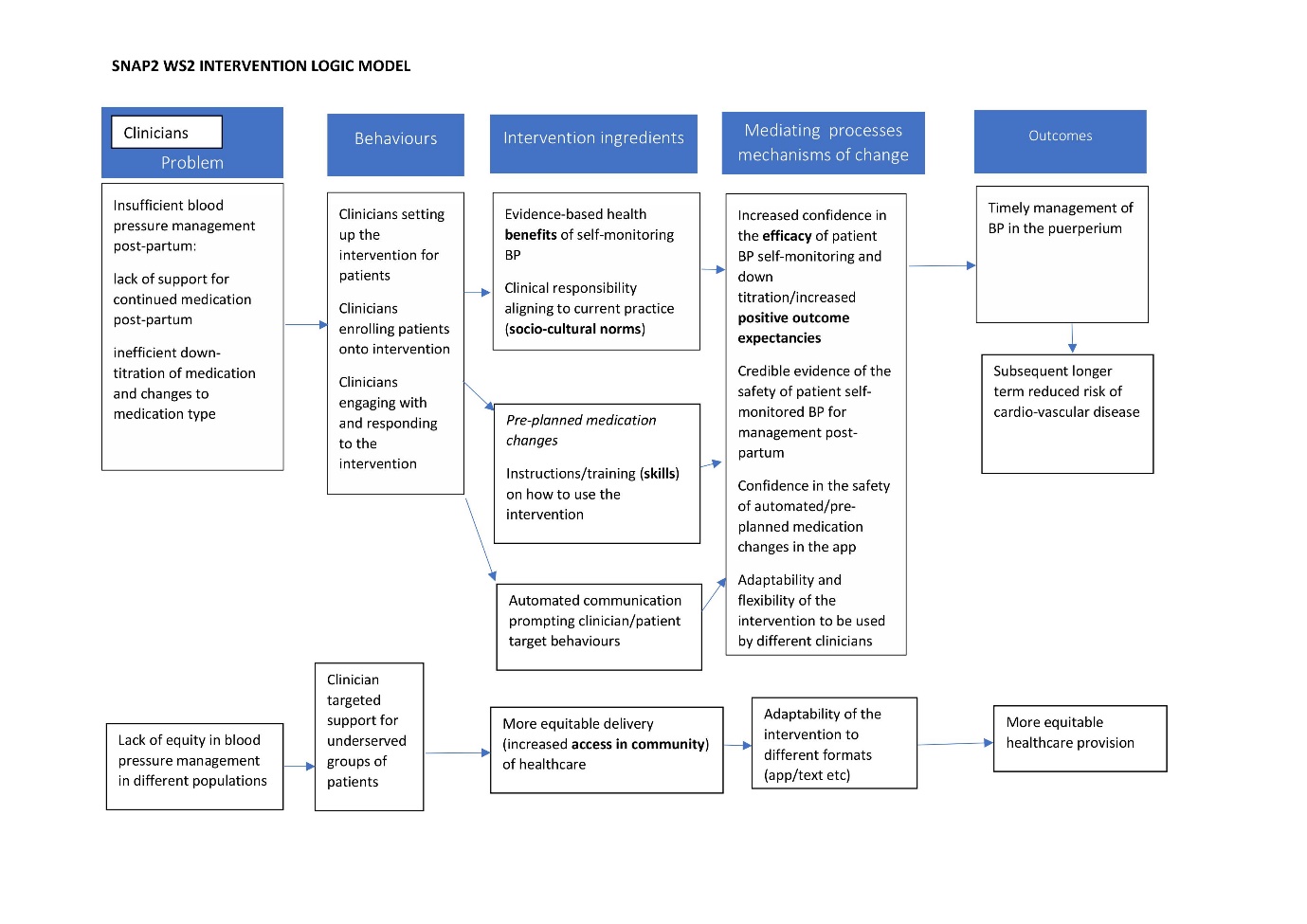

Supplement: online supplemental file 1 [file bmjopen-15-6-s001.docx]

**Supplementary file 10**

**Screenshots of the patient-facing elements of the digital intervention**


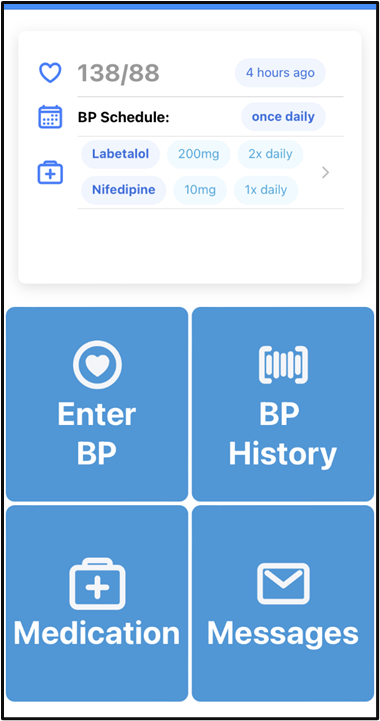

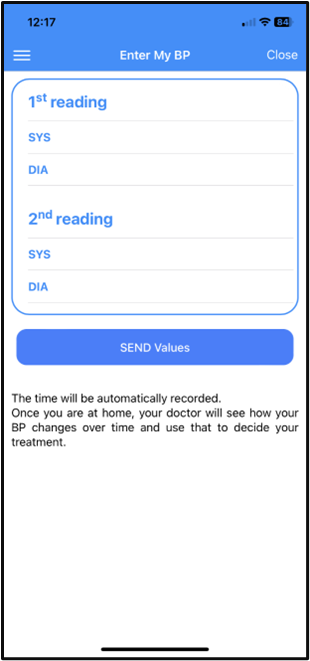

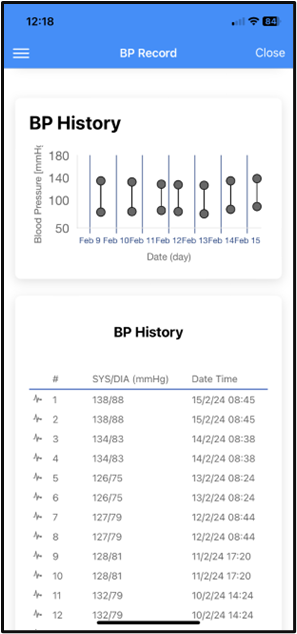

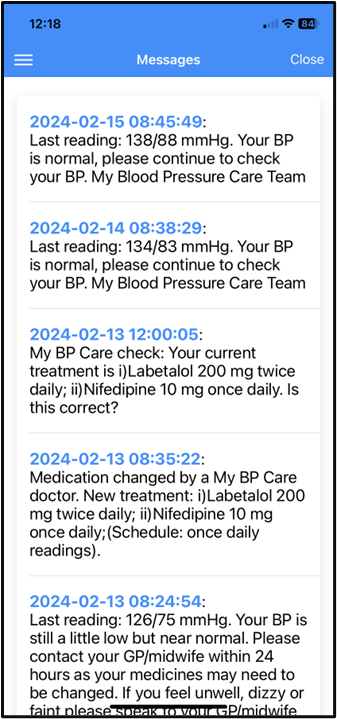

Supplement: online supplemental file 10 [file bmjopen-15-6-s010.docx]
