# Supplementary material for: Using qualitative research and the person-based approach to coproduce an inclusive intervention for postpartum blood pressure self-management
Source: BMJ Open. 2025 Jun 24;15(6):e098162. doi: 10.1136/bmjopen-2024-098162 (PMC12198848; doi:10.1136/bmjopen-2024-098162)
Supplement: online supplemental file 2 [file bmjopen-15-6-s002.docx]

***Supplementary File 2***

*Intervention planning table*

| **Target behaviour** | **Barrier/ *facilitator* to target behaviour** | **Detail about the evidence for barrier/ facilitator/ intervention ingredient** | **Possible intervention ingredient to promote facilitators and overcome barriers** |
| --- | --- | --- | --- |
| Accepting the intervention | *Barriers*   - Patients may not trust an automated system as compared to a physical clinician   *Facilitators*   - Having a plan to manage their BP/ Knowing what to do when at home - Feeling empowered to participate in one’s BP management - Convenience of self-managing from home - Easy and universally applicable patient recruitment | PPI  PPI  Thomas, Drewry [1] study on patient perceptions, opinions and satisfaction of telehealth with remote blood pressure monitoring postpartum  Thomas, Drewry [1] study on patient perceptions, opinions and satisfaction of telehealth with remote blood pressure monitoring postpartum and Demers and Wagner [2] study on nurses’ and physicians’ perspectives on text-based postpartum blood pressure monitoring.  Stakeholders meeting and Hauspurg, Lemon [3] study on racial differences in postpartum blood pressure trajectories among women after a hypertensive disorder of pregnancy. | - Reassuring the patients that the medication changes have been prepared by their obstetrician and that a clinician would look at their record when required - Highlighting that the intervention empowers them for self-management - Practical training on BP self-monitoring before discharge - Ensuring intervention is simple and easy to use by different patients in different homes   Ensuring the recruitment strategy is pragmatic to all obstetricians and patient demographics |
| Downloading the intervention | *Barriers*   - Technology problems (old phone, no memory to support the intervention) - Language barriers for non-English speakers   *Facilitators*   - Having access to the intervention | Stakeholder-research team  Stakeholder-research team  Triebwasser, Janssen [4] study on implementation of text-based blood pressure monitoring for postpartum hypertension- patients enrolled into the platform before discharge | - Having options for alternative intervention format e.g. website, or text message - Having the option to provide patients with a smart phone to download the intervention - Having family that speak English being present when explaining the intervention to patients - Clinicians assisting the patients to download the intervention before discharge |
| Patients BP self-monitoring | *Barriers:*   - Lack of time to monitor as prioritising baby - Feeling too tired to monitor - Not feeling sick/feeling fine-not seeing the need for self-monitoring - Lack of self-efficacy for BP self-monitoring - Patients feeling unable to take up lifestyle changes post-partum - Patients forgetting or not being consistent with their BP measuring and recording   *Facilitators:*   - Self-efficacy for BP self-management - Belief in the importance of BP monitoring postpartum - Access to a validated BP monitor for home use - Intervention fitting into their lifestyle - Low cost | PPI, and Thomas, Drewry [1] study on patient perceptions, opinions and satisfaction of telehealth with remote blood pressure monitoring postpartum, and Cairns, Tucker [5] SNAP HT Trial on self-management of post-natal hypertension  PPI  Band, Hinton [6] intervention development for early detection of raised BP in pregnancy and Hinton, Hodgkinson [7] study of clinicians’ perceptions of introducing home monitoring of BP in pregnancy into maternity care  PPI and stakeholders’ group  Stakeholders group and Triebwasser, Janssen [4] study on implementation of text-based blood pressure monitoring for postpartum hypertension.  Band, Hinton [6] intervention development for early detection of raised BP in pregnancy and Hinton, Hodgkinson [7] study of clinicians’ perceptions of introducing home monitoring of BP in pregnancy into maternity care  PPI and Hauspurg, Lemon [3] study on racial differences in postpartum blood pressure trajectories among women after a hypertensive disorder of pregnancy.  PPI  PPI and Triebwasser, Janssen [4] study on implementation of text-based blood pressure monitoring for postpartum hypertension and Hoppe, Williams [8]. Study on telehealth with remote blood pressure monitoring for postpartum hypertension and Hauspurg, Lemon [3] study on racial differences in postpartum blood pressure trajectories among women after a hypertensive disorder of pregnancy.  Thomas, Drewry [1] study on patient perceptions, opinions and satisfaction of telehealth with remote blood pressure monitoring postpartum  Triebwasser, Janssen [4] study on implementation of text-based blood pressure monitoring for postpartum hypertension. | - Making the monitoring as easy and quick as possible - Reminding the patients the importance of monitoring - Giving patients credible and persuasive information on benefits of self-monitoring BP and the highlighting that BP problems can often be asymptomatic - Practical training on BP self-monitoring before discharge, intervention allowing the patients to practise using it before discharge - The option for on-going support with the self-monitoring - Re-assuring patients that the intervention will not involve major lifestyle change like exercise and diet - Having a prompt and reminders on the intervention for whenever the patients need to take the BP measures or when they forget - Practical training on BP self-monitoring before discharge, intervention allowing the patients to practise using it before discharge - The option for on-going support with the self-monitoring - Giving patients credible and persuasive information on benefits of self-monitoring BP - Providing a validated BP monitor for patients to use - Making the intervention easy to incorporate into the patients daily life - Making the intervention and blood pressure monitor free for patients |
| Patient in-putting BP entries in intervention | *Barriers*   - Patients may make errors in in-putting their BP measure - Patients worrying about their privacy   *Facilitators*   - Patients feeling confident to use the intervention | Stakeholder-research team  Hauspurg, Lemon [9] study on postpartum remote hypertension monitoring implemented at the hospital level  Stakeholder-research team and Triebwasser, Janssen [4] study on implementation of text-based blood pressure monitoring for postpartum hypertension. | - The intervention may be programmed for patients to take a photo of their BP readings or the intervention could be synchronised with the BP monitor so that it automatically records from the monitor - Reassuring information on the data security of the intervention - Practical training on BP self-monitoring before discharge, intervention allowing the patients to practise using it before discharge |
| Patients continuing post-partum medication as required | *Barriers:*   - Concerns about the medication not being suitable for breast-feeding - Forgetting to take the medication - Not feeling like they need the medication - Feeling medicalised - Patients being confused on what to take due to lots of medication changes post-partum - Patients not being open about their diagnosis and struggling with taking medication around their wider circles (work, family and friends) - Patients running out of medication   *Facilitators:*   - Wanting more knowledge on elevated BP post-partum and how to handle it - Concerned about developing chronic hypertension | Stakeholders- research team  PPI  PPI  Cairns, Tucker [5] SNAP HT Trial on self-management of post-natal hypertension  PPI  PPI  Stakeholders group  PPI  PPI | - Giving patients credible information affirming that their prescribed medication will be suitable for breast-feeding - The patient could have an alarm set to remind them to take the medication - Giving patients credible and persuasive information on benefits of and risks of not taking the right medication for blood pressure - Reassuring patients of the importance of well-managed BP for their holistic health at present and in the future - Having clear written instructions of when and how to take each medication on the intervention - Designing the intervention in friendly language so that the patients feel free to ask for support from different providers if they are struggling and assuring them that HDPs are not uncommon and many patients need medication to manage them - The intervention ensuring that patients are discharged with two weeks’ worth of medication and their prescription being sent to their GP upon discharge - Giving credible and persuasive information on importance of managing blood pressure post-partum - Giving persuasive information on expected outcomes and benefits of good blood pressure management post-partum |
| Patients adhering to medication change advice | *Barriers*:   - Not understanding the importance of effective BP medication down titration post-partum - Finding the suggested medication changes difficult to understand - Lacking confidence in the suggested medication changes on the intervention - Wanting a clinician to communicate directly to them about medication changes instead of a notification on the intervention   *Facilitators:*   - Confidence that the medication change is from a clinician who knows their medical history | PPI  PPI  PPI  PPI and Triebwasser, Janssen [4] study on implementation of text-based blood pressure monitoring for postpartum hypertension.  PPI  Triebwasser, Janssen [4] study on implementation of text-based blood pressure monitoring for postpartum hypertension. | - Giving credible evidence of the benefits of effective down-titration of medication post-partum - Having clear written instructions on the intervention of when and how to change the BP medication - Reassuring the patients that the medication changes have been prepared by their doctor - Explaining that through the intervention, the clinicians will be able to see their BP histories and hence tailor medication changes to that |
| Clinician responding to intervention flag based on patient BP recordings | *Barriers*   - The clinicians may not respond to their flags on the intervention   *Facilitators*   - The intervention to have a drop down menu of medication already listed | Stakeholders’ group  Stakeholder-research team | - Ensure that clinicians understand their role in managing the flags relating to patient BPs on the intervention - Making the intervention as easy as possible for the clinicians to make the medication changes |
| Clinician supporting continued patient BP self-management | *Barriers:*   - Lack of continuity of care in the puerperium period - Lack of clinical capacity to support the self-management and medication changes ?particularly in the postnatal period   *Facilitators:*   - Patient self-monitoring frees up clinical time that would have been spent monitoring patients - Self-management of BP not yet standard practice in all healthcare setting | Stakeholders’ group and  Cairns, Tucker [5] SNAP HT Trial on self-management of post-natal hypertension  Stakeholders’ group and Triebwasser, Janssen [4] study on implementation of text-based blood pressure monitoring for postpartum hypertension.  Stakeholder-research team  Hinton, Hodgkinson [7] study of clinicians’ perceptions of introducing home monitoring of BP in pregnancy into maternity care  Hinton, Hodgkinson [7] study of clinicians’ perceptions of introducing home monitoring of BP in pregnancy into maternity care | - Ensuring the intervention can be used by different clinicians including doctors, nurses, midwives, health visitors, pharmacists etc - Intervention making clinical work easier by delegating monitoring to the patient   • Intervention making clinical work easier by delegating monitoring to the patient   - Ensure self management of BP is easy to integrate into current practice |
| Clinician supporting patient self-monitored BP for medication changes | *Barriers:*   - Lack of clear plan for which clinician will be caring for the patient while on intervention - Different care pathways in different locations within the puerperium period - Patients not attending their post-natal BP appointments if they are made - Uncertainty about the accuracy of patient self-monitored BP (reliability of the machines, patient skill, appropriate cuff sizes) - Uncertainty about the safety of patient self-managed BP - Potential increase in clinical workload (multiple readings in short period of time) - Different members of the clinical team not being aware of the intervention or how to engage with it   *Facilitators:*   - Chance to empower the patients - Lack of clinical capacity for home visits - Aligning with current practice - Enhancing patient safety - Intervention providing quick accessible information to clinicians | Stakeholders’ group  Stakeholders’ group and Triebwasser, Janssen [4] study on implementation of text-based blood pressure monitoring for postpartum hypertension.  Hauspurg, Lemon [9] study on postpartum remote hypertension monitoring implemented at the hospital level  Demers and Wagner [2] nurses’ and physicians’ perspectives on text-based postpartum blood pressure monitoring.  Cairns, Tucker [5] SNAP HT Trial on self-management of post-natal hypertension and Demers and Wagner [2] nurses’ and physicians’ perspectives on text-based postpartum blood pressure monitoring.  Hinton, Hodgkinson [7] study of clinicians’ perceptions of introducing home monitoring of BP in pregnancy into maternity care and Stakeholders group  Stakeholders group  Thomas, Drewry [1] study on patient perceptions, opinions and satisfaction of telehealth with remote blood pressure monitoring postpartum  Stakeholders group  Stakeholders -research team  Stakeholders- research team and Hauspurg, Lemon [9] study on postpartum remote hypertension monitoring implemented at the hospital level  Stakeholders- research team and Triebwasser, Janssen [4] study on implementation of text-based blood pressure monitoring for postpartum hypertension. | - Identifying different clinicians who will be able to support the intervention in different locations including doctors, midwives, nurses, pharmacists and health visitors - Patients being able to self-monitor at home - Highlighting that self-monitoring is a valid form of clinical monitoring - Ensuring patients are trained in self-monitoring and provided with validated BP machines and their correct cuff sizes - Credible evidence of the safety of patient self-monitored BP for management post-partum - Clinicians will act based on flags (generated by an alogorithm) on the intervention rather than individual submitted BP readings - Providing training to different clinicians about how to engage with the intervention - Patients having an active role in self-monitoring and management of their BP - Patient self-monitoring will remove the need for clinician home visits - Ensure feedback to self-monitored results is automated for timely action - Having a summary screen with BP trends and current medication that the patients can show each clinician if required - The patient’s clinicians will have access to their own dashboard on the intervention containing patient details |
| Handling patient deterioration | *Barrier*   - Telling the patient to call their GP may take too long for them to access care   *Facilitators*   - The intervention having direct contact with the patient’s clinician | Stakeholders’ group  Stakeholders’ group | - The intervention will email the patients’ clinician directly with suggested actions to take to manage the patient. The patient will simultaneously be advised to contact their clinician for medical care including out of hours contacts |
| Clinician targeted support for underserved groups of patients | *Barriers:*   - Not hearing the voices of underserved groups - Lack of access and/or ability to use technology eg internet, smart phones or home computers/tablets - Language barrier - Patients being too scared to voice their struggles for fear of baby being taken away - Lack of trust in clinicians (among underserved communities)   *Facilitators:*   - Knowing that ethnic minorities particularly black patients have worse BP outcomes | Stakeholders-research team and Cairns, Tucker [5] SNAP HT Trial on self-management of post-natal hypertension  Stakeholders-research team and Demers and Wagner [2] nurses’ and physicians’ perspectives on text-based postpartum blood pressure monitoring and Triebwasser, Janssen [4] study on implementation of text-based blood pressure monitoring for postpartum hypertension and  and Hauspurg, Lemon [3] study on racial differences in postpartum blood pressure trajectories among women after a hypertensive disorder of pregnancy.  Stakeholders-research team  Stakeholders-research team  PPI  Stakeholders-research team  PPI and Hauspurg, Lemon [3] study on racial differences in postpartum blood pressure trajectories among women after a hypertensive disorder of pregnancy | - Intervention giving patients the opportunity to share their symptoms remotely. After further discussions with stakeholders, it was decided that asking patients their symptoms might cause anxiety and confusion rather than facilitating self-monitoring. - Designing the Intervention in different forms eg text message instead of the intervention and/or providing on-going support with the self-monitoring - Allowing for patient’s family that speak English be present (with the patient’s permission) during training of the intervention to provide translation to the patient - Reassuring information that the clinicians and social services are there to help - Reassuring information on the need for and efficacy of BP self-monitoring post-partum among all communities including black patients |

**References**

1. Thomas, N.A., et al., *Patient perceptions, opinions and satisfaction of telehealth with remote blood pressure monitoring postpartum.* BMC Pregnancy and Childbirth, 2021. **21**: p. 1-11.

2. Demers, S. and J.M. Wagner, *Nurses’ and physicians’ perspectives on text-based postpartum blood pressure monitoring.* The Journal for Nurse Practitioners, 2021. **17**(3): p. 348-353.

3. Hauspurg, A., et al., *Racial differences in postpartum blood pressure trajectories among women after a hypertensive disorder of pregnancy.* JAMA network open, 2020. **3**(12): p. e2030815-e2030815.

4. Triebwasser, J.E., et al., *Successful implementation of text-based blood pressure monitoring for postpartum hypertension.* Pregnancy Hypertension, 2020. **22**: p. 156-159.

5. Cairns, A.E., et al., *Self-management of postnatal hypertension: the SNAP-HT trial.* Hypertension, 2018. **72**(2): p. 425-432.

6. Band, R., et al., *Intervention planning and modification of the BUMP intervention: a digital intervention for the early detection of raised blood pressure in pregnancy.* Pilot and Feasibility Studies, 2019. **5**: p. 1-12.

7. Hinton, L., et al., *Exploring the potential for introducing home monitoring of blood pressure during pregnancy into maternity care: current views and experiences of staff—a qualitative study.* BMJ open, 2020. **10**(12): p. e037874.

8. Hoppe, K.K., et al., *Telehealth with remote blood pressure monitoring for postpartum hypertension: a prospective single-cohort feasibility study.* Pregnancy hypertension, 2019. **15**: p. 171-176.

9. Hauspurg, A., et al., *A postpartum remote hypertension monitoring protocol implemented at the hospital level.* Obstetrics & Gynecology, 2019. **134**(4): p. 685-691.
