# Supplementary material for: Using qualitative research and the person-based approach to coproduce an inclusive intervention for postpartum blood pressure self-management
Source: BMJ Open. 2025 Jun 24;15(6):e098162. doi: 10.1136/bmjopen-2024-098162 (PMC12198848; doi:10.1136/bmjopen-2024-098162)
Supplement: online supplemental file 3 [file bmjopen-15-6-s003.docx]

***Supplementary File 3***

*Table of Changes excerpt*

| Intervention page | Source | Date received | Negative comments | Positive comments | Neutral comments | Possible change | Reason for change | MoScow Must do; Should do; Could do; Would Like to do | Changed or not changed |
| --- | --- | --- | --- | --- | --- | --- | --- | --- | --- |
| All | Stakeholders' meeting | 27/02/2023 | Language barrier a major problem in NHS app (CR), Best practice is to translate app (LY) |  |  | Make app compatible with Recite Me and other translating apps (CR), App to contains lots of infographics (LY)) | REP, IMP, EXP (PPI and stakeholders), To make the app more accessible to those who are not fluent English speakers as well as those who are English speakers but not very literate. | Would like to do | C |
| Home page | Stakeholders' meeting | 27/02/2023 |  |  | the summary page should be available to both patients and clinicians | Include summary page in both clinician and patient portal | REP, EAS, IMP to enhance ease of use | Must do | C |
| Med plan page | Stakeholders' meeting | 27/02/2023 |  |  | the medication changes plan should be contained in that page. | Include medication change plan in the app | EXP (clinicians especially GPs appreciate having a guide on how to prescribe post-partum women) | Could do | NC |
| Med plan page | Stakeholders' meeting | 27/02/2023 |  |  | to include the option of having a clinician declining the medication suggested and updating with the one they prescribe | Include option for clinician to change medication | REP, EXP(clinician highlighted that sometimes other clinicians change patient medication different from prepared medication plan | Must do | C |
| BP record page | Stakeholders' meeting | 27/02/2023 |  | Liked the visualisation of the BP history |  | line graphs-libre graphs would work better with the two readings in the graph as well as what normal readings would be. Stakeholder also asked if it was possible to plot on the graph the medication taken so that people could follow the blood pressure changes alongside medication | EXP (clinicians found the visual depiction easy and quick to understand) | Should do | C |
| More info page1 | Stakeholders' meeting | 27/02/2023 |  |  | Need more info on BP levels | Include a table of BP groupings and alert messages on what patient should do at each level e.g. contact their clinician | REP, EXP (clinicians said the more info was important for patient information) | Must do | C |
| Intro page | Stakeholders' meeting | 27/02/2023 | The graphic at the beginning of the woman holding the baby might be distressing to those who may not have had a live birth. |  |  | Possible Icon change | NCON, further research found it not distressing | Could do | NC |
| Home page | Stakeholders' meeting | 27/02/2023 |  |  | Include brief patient diagnosis and treatment | Include patient diagnosis, patient medical history/other medication, date of delivery and calculated post-natal days on Home page | EXP (clinicians said they would appreciate a brief background of patient diagnosis for quick access) | Must do | C |
| More info page2 | Stakeholders' meeting | 27/02/2023 |  |  | Include links to other resources patients can draw upon | Include more links for information like NHS website pages, Action on Pre-eclampsia and the Motherhood group. |  | Should do | C |
|  |  |  |  |  |  |  |  |  |  |
| Clinician emails | Think-aloud- obs |  |  |  | Knowing when (or capturing the info when) a patient hasn't taken their medication (especially when BP raised/high/very high. Then including this info in the clinician messages. | Ask participants a question in addition to the third reading similar to 'When was the last time you took your anti-hypertensive medication ?(options: in the past 4h, in the past 24h, or more than 1 day ago)" |  | Could do | NC |
