# Supplementary material for: Using qualitative research and the person-based approach to coproduce an inclusive intervention for postpartum blood pressure self-management
Source: BMJ Open. 2025 Jun 24;15(6):e098162. doi: 10.1136/bmjopen-2024-098162 (PMC12198848; doi:10.1136/bmjopen-2024-098162)
Supplement: online supplemental file 4 [file bmjopen-15-6-s004.docx]

**Supplementary file 4**

**Patient demographic details**

*Table 3- former patient participants*

| Participants | Ethnicity | Highest education qualification | Employment status | Religion | Home Index of deprivation |
| --- | --- | --- | --- | --- | --- |
| Pt1 | Black British | National Vocational Qualification | Unemployed | Christian | High deprivation |
| Pt2 | Black African | National Vocational Qualification | Employed | Christian | High deprivation |
| Pt3 | Asian | Secondary school | Unemployed | Muslim | High deprivation |
| Pt4 | Black British | National Vocational Qualification | Employed | Christian | High deprivation |
| Pt5 | British Asian | National Vocational Qualification | Unemployed | Muslim | High deprivation |
| Pt6 | British Asian | Masters’ degree | Unemployed | Muslim | High deprivation |
| Pt7 | White British | Bachelor’s degree | Employed | Not religious | Not disclosed |

Patient demographic details for Phase 3 follow-up semi-structured interviews

*Table 4- patient participants*

| Patients | Ethnicity | Highest education qualification | Employment status | Home Index of deprivation |
| --- | --- | --- | --- | --- |
| Pts1 | White Other | University degree | Unemployed | Low deprivation |
| Pts2 | Black African | University degree | Employed | High deprivation |
| Pts3 | White other | University degree | Employed | Moderate deprivation |
| Pts4 | White British | Secondary school | Employed | High deprivation |
| Pts5 | British Asian | University degree | Employed | High deprivation |
| Pts6 | Black African | University degree | Employed | High deprivation |
| Pts7 | Black African | National Vocational Qualification | Employed | High deprivation |
| Pts8 | White British | National Vocational Qualification | Employed | High deprivation |
| Pts9 | White British | University degree | Employed | Moderate deprivation |
| Pts10 | White British | University degree | Employed | Moderate deprivation |
| Pts11 | Black African | University degree | Employed | High deprivation |
| Pts12 | White other | University degree | Employed | Moderate deprivation |
| Pts13 | Black African | Secondary school | Unemployed | High deprivation |
| Pts14 | Black British | National Vocational Qualification | Unemployed | High deprivation |
| Pts15 | Asian | Secondary school | Employed | High deprivation |
| Pts16 | White British | University degree | Employed | Moderate deprivation |
| Pts17 | White British | University degree | Unemployed | High deprivation |
| Pts18 | White other | University degree | Employed | Moderate deprivation |
| Pts19 | White British | University degree | Employed | Moderate deprivation |
| Pts20 | White British | University degree | Employed | Moderate deprivation |
| Pts21 | Asian | University degree | Employed | High deprivation |
| Pts22 | White British | National Vocational Qualification | Employed | High deprivation |
| Pts23 | White British | University degree | Employed | Not disclosed |
