# Supplementary material for: Using qualitative research and the person-based approach to coproduce an inclusive intervention for postpartum blood pressure self-management
Source: BMJ Open. 2025 Jun 24;15(6):e098162. doi: 10.1136/bmjopen-2024-098162 (PMC12198848; doi:10.1136/bmjopen-2024-098162)
Supplement: online supplemental file 5 [file bmjopen-15-6-s005.docx]

**Supplementary file 5- interview quotes**

Phase 2: Quotes from the patient think alouds

| Theme | Quote |
| --- | --- |
| Intervention purpose and name | Pt5: you have a really good thing…that's why I'm surprised because at that time [post-pregnancy], my midwife not even give me any kind of suggestions… Just give me a flyer. So I was scared at that time… You guys doing a really, good thing. In future, the girls need these kind of stuff. So we can just check [BP] at home. |
| Intervention clarity and attractiveness | Pt7: Yeah, that seems clear enough. Yes, that's good, because I think it helps people to understand future health problems, because that's what I was worried about that you just think ohh the placenta is gone. Yes, it's got your medications and your dosage. That's a nice visual and is the like little green heart with the blood pressure reading.  Pt3: it's just so clear… people can see the thing before even reading.  Pt5: It's[leaflet] beautiful that a lot of information inside, especially with the colours, you know that we understand this like my kind of people who are not too much educated. Ohh red it means there is something dangerous in here. Yellow, it's little bit dangerous and the blue is different. Umm, so that's good. |
| Reassurance of clinical oversight | Pt7: Medicines you take, so we can take medicine without doctor prescription? Or is the doctor that has put the prescription on there. Important to have doctor knowing. |
| Reassurance of medication safety | Pt1: I'll be very concerned and if you gave me prescription medication, I'm worried to take it. Because I'll be concerned of the effects that it's having on my baby cause everything that goes into our bodies goes to your breastfeeding baby. |
| Need for reminders | Pt4: If they haven't done it by a certain time, say 3:00 o'clock of an afternoon trigger the app to say, can you please put your reading in for today. |

Phase 3: Quotes from the semi-structured interviews with patients

| Theme | Quote |
| --- | --- |
| Intervention clarity | Pts1: The App was straight-forward and easy to use.  Pts14: The messages were perfect, straightforward.  Pts22: Well, as you can understand my English is not well, but I understand everything in the app. So I don't think that the language needs to be changed. |
| Patient training | Pts5: And one of the midwives showed me, you know what the app looked like, so I remembered because she came to see me in hospital.  Pts11: Research midwives are quite thorough and given the information and they told me what it was like, what I was supposed to do. |
| Medication management | Pts12: I changed it and wrote no medication, stopped medication. It's not confusing at all.  Pts14: I've been waiting for a doctor [GP], however, I can't get an appointment. |
| Feedback on BP readings | Pts 6: It is quite nice message because when they [BP] are quite high. They'll tell you. Maybe what to do or what not to do.  Pts 15: And that was something that I did like I every time I've recorded my blood pressure, there was a message kind of an automated message just to tell me how I'm going. It's it reassuring as well … to have those messages, it does help. It's almost like someone's telling you, giving you the advice as opposed to you making your own decision. |
| Reassurance of clinical oversight | Pts 21: I thought it was really good just because I was told that they wanted to, even with the enalapril, they wanted to stop my medication. But I was really worried because of like, risk of post-partum pre-eclampsia. And so it was good to enter the numbers and someone like a health care professional will look at it. |
| Instant feedback based on BP reading | Pts 16: I was actually feeling quite lightheaded… but I went to see my GP and they halved my medication and then said that when it would be normal for a few days I could stop… like with it being Bank Holiday Monday and my blood pressure being low, I made the decision on my own to hold my medication that day. |
| Easily accessible BP record | Pts16: When I originally contacted the GP, it was through the contact form online. And so I uploaded a photograph of that page with all my blood pressures. So when I actually went in, he actually didn't even take my blood pressure because I think he obviously had all the information on the trends before.  Pts11: I have seen my GP last week. To show that it [BP history] was there. Yeah, because it was very useful because it wasn't just that one time reading. I thought, this is what I'm doing and this is the record… The past two weeks, so that actually prompted her to stop the medication twice a day. So that's quite useful.  Pts6: So you can basically see like over time days that your blood pressure was may be very good or very high. So at least that's help for somebody. If maybe after a period of time... If your BP has been high for quite a while, maybe seek medical advice |
| Challenges to and strategies for adhering to the intervention | Pts17: So it's [self-monitoring] easy if you don't have a baby, but with a newborn baby. It's a bit tricky with the hours to manage to do it.  Pts13: I wake up in the morning, I brush my teeth. I take my medication and I go for the machine to check and sometime I just take the machine to check then take breakfast.  Pts21: Like I would put the monitor. It sounds really silly, but I put that next to my babies nappies. So when I would wake up in the morning to change her then see the monitor, OK let me check my blood pressure first before I change her now so that was something that would like prompt me to like check my blood pressure.  Pts8: Yeah, but it only gives you a reminder after 24 hours from your last reading. So it should probably be about 12. So you can submit within the day. |
| Desire for two way communication between patients and clinicians | Pts5: Further, it wasn't a two way, message service, so I can't put any messages up to say if I was having issues or anything I would have to phone up and if I was having any issues. And so I thought maybe it would be helpful to have it. |

Phase 3: Quotes from semi-structured interviews with clinicians

| Theme | Quote |
| --- | --- |
| Patient recruitment into the intervention | Mdwf3: So when people realise that they're gonna get a BP monitor that they've been told they need one anyway, usually by another clinician that they thought they were gonna have to buy. And then when they realized ohh I can do this study and I'm gonna get one. That's quite a good selling point.  Mdwf2: the ease of adding women to the app and making a profile for them, that was good, that was fairly straightforward and women seemed quite eager to use the app as well. |
| Patient training | Mdwf3: We actually put it [BP monitor] on them, we actually run it through and they take a blood pressure, they had to do it because it's a new machine and we just do kind of a dummy run in the hospital… I say download it [intervention] now …So I can show you what it looks like.  Mdwf1: I don't know how long I'll be able to spend with each particular participant, and then it, you know, relying on the Wi-Fi to be working in the hospital to download the app, which proved a problem. And the hospital Wi-Fi here isn't brilliant. |
| Reassurance of clinical oversight | Mdwf1: I mean, a lot of the ladies liked the knowledge that I was looking at the app daily. I made clear that it was not 24 hour seven days a week service … it's very good because…it prevents them having to call me and they like to know that I'm kind of remotely caring for them. And they are not on their own. A lot of the ladies like the fact that they don't have to ring triage…But if they have the app they they're like submitting because it's kind of that remote check in.  Obs1: My main issue was when I got the email it obviously emailed out several of us and when I went on to the app I couldn't tell if someone had actioned it or not… I had to send an email out round to people to say, ‘ Has this been done or do you need me to do something?’ It'd be nice if there's an easy way of finding out right, this has been actioned. |
| Medication management | Obs1: I think reminding women that part of the onus is on them to get that followed up that medication… needs to be spelt out whether that's included in a message on the app. As a FYI. |
