# Supplementary material for: Using qualitative research and the person-based approach to coproduce an inclusive intervention for postpartum blood pressure self-management
Source: BMJ Open. 2025 Jun 24;15(6):e098162. doi: 10.1136/bmjopen-2024-098162 (PMC12198848; doi:10.1136/bmjopen-2024-098162)
Supplement: online supplemental file 6 [file bmjopen-15-6-s006.docx]

**My BPCare Patient intervention development think-aloud interview schedule**

1. **INTRODUCTION**

- Remind the participant that the interview is to find out their thoughts of the intervention element.
- Check if they have read the information sheet and completed the consent form.
- Ask them if they have any questions.
- Check if they are still happy to be interviewed and remind them that they can stop at any time if they wish to.
- Double check that they are ok for the interview to be recorded reminding them that the data will be anonymised. If yes, start recording.

1. Could you tell me about the pregnancy where you had blood pressure problems? [Prompts- how long ago, how were you diagnosed, were you given medication before delivery?]
2. What happened after the pregnancy ended? [Prompts- did the high blood pressure continue, what did the doctor/midwife at the hospital tell you? Did you get to see your GP once you were home?]
3. Were you on medication after the delivery? Did you stop medication at some point? [if yes, how did you stop? Did a doctor/nurse/midwife tell you to stop?]
4. What did you think of that experience?

- We are looking to develop tools to help people manage their blood pressure after pregnancy. We have developed an App where women can record their blood pressure once they leave the hospital. Patients will be given a blood pressure monitor and asked to record their blood pressure everyday for the first few weeks. We will also send them messages and reminders to encourage them to record their blood pressure. Today I will be showing you the App and messages.
- We are interested in your thoughts as you go through them. Please speak-aloud what you are seeing and what you are thinking. Please do feel free to say any negative thoughts you may have about the content as these will be really useful in helping us to improve it. If you think anything is confusing or unclear, please also say that out loud. Feel free to be brutally honest.
- I will be silent as you go through it just so that I don’t interrupt your thoughts but we will chat some more at the end.

1. **THINK ALOUD AND RESEARCHER PROMPTS**

- [if needed] What are you looking at?
- What have you clicked on?
- What are your thoughts on that?

1. **POST THINK ALOUD QUESTIONS**

- Overall, what do you think about the whole idea? App? Messages?
- Can you tell me about anything you thought needs to be changed to improve it? [Probe- anything we can do to make it better or easier for you to use it if you needed to?]
- At the time you had the blood pressure in pregnancy, how would you have found using this?
- Anything that I have forgotten to ask that you would like to share about this?
- Collect demographic details (Age, ethnicity, education qualifications, employment status, post-code)

**Thank you so much that is really helpful, we really appreciate it. I will send you the £20 shopping voucher as a thank you for your time. Have a lovely day**
