# Supplementary material for: Using qualitative research and the person-based approach to coproduce an inclusive intervention for postpartum blood pressure self-management
Source: BMJ Open. 2025 Jun 24;15(6):e098162. doi: 10.1136/bmjopen-2024-098162 (PMC12198848; doi:10.1136/bmjopen-2024-098162)
Supplement: online supplemental file 7 [file bmjopen-15-6-s007.docx]

**My BPCare Clinician intervention development think-aloud interview schedule**

1. **INTRODUCTION**

- Remind the clinicians that the interview is to find out their thoughts of the intervention element.
- Check if they have read the information sheet and completed the consent form.
- Ask them if they have any questions.
- Check if they are still happy to be interviewed and remind them that they can stop at any time if they wish to.
- Double check that they are ok for the interview to be recorded reminding them that the data will be anonymised. If yes, start recording.

1. Perhaps we can start by you telling me a bit about your role [Prompt- job role, length of service, institution]

- This is a study to develop an intervention for self-management of blood pressure post-partum. The intervention will involve patient components and clinician components. The patient component involves an App, blood pressure monitor and motivating messages promoting their self-management. The clinician components involve a clinician dashboard where they will be able to see the patient’s BP record and medication, template clinician emails to facilitate clinician-clinician communication and management of patient and an advice document containing the latest NICE guidance on the management of BP post-partum. Today I will be showing you some of what we have developed so far on the clinician components.
- We are interested in your thoughts as you go through the content. Please speak-aloud what you are seeing and what you are thinking. Please do feel free to say any negative thoughts you may have about the content as these will be really useful in helping us to improve it. If you think anything is confusing or unclear, please also say that out loud.
- I will be silent as you go through the content so that I don’t interrupt your thoughts but we can chat some more when you are done.

1. **THINK ALOUD AND RESEARCHER PROMPTS**

- [if needed] What are you looking at?
- What have you clicked on?
- What are your thoughts on that?

1. **POST THINK ALOUD QUESTIONS**

- Overall, what do you think about the content?
- Can you tell me about anything you thought needs to be changed to improve it? [Probe- any ideas on how we can improve it?]
- How do you think you would find using this?
- Anything that I have forgotten to ask that you would like to share about this?

**Thank you so much and have a lovely day**
