# Supplementary material for: Using qualitative research and the person-based approach to coproduce an inclusive intervention for postpartum blood pressure self-management
Source: BMJ Open. 2025 Jun 24;15(6):e098162. doi: 10.1136/bmjopen-2024-098162 (PMC12198848; doi:10.1136/bmjopen-2024-098162)
Supplement: online supplemental file 8 [file bmjopen-15-6-s008.docx]

**My BPCare intervention development clinician Interview/focus group topic guide**

**Clinician telephone interview schedule**

- Remind the clinicians that the interview is to find out their experience of the intervention.
- Check if they have read the information sheet and completed the consent form.
- Ask them if they have any questions.
- Check if they are still happy to be interviewed and remind them that they can stop at any time if they wish to.
- Double check that they are ok for the interview to be recorded reminding them that the data will be anonymised. If yes, start recording.

**Introduction**

1. How have you found MyBPCare so far? Overall what would you say went well? What did not go well? General lessons learnt for the next stage?

**Intervention**

1. What do you think we could add to the initial clinician training to promote success of MyBPCare? What should we highlight as the key take home messages?
2. What advice would you give to someone beginning recruitment for MyBPCare? Probes: Timing of recruitment? Patient training? Set-up on the dashboard? Follow-up? How to handle discharge?
3. In your opinion, how can we promote patients recording their BP daily on MyBPCare?
4. How was medication change handled clinically at your site? How does this compare with usual care? How could we promote patient’s updating their medication record of the App?
5. How were the emails communication about patients handled? Would this be compatible with usual practice? If not how could this part of MyBPCare be managed in usual practice?

**Transition to primary care**

1. How does your site handle the transition to primary care? Were you able to discharge the patients to their GP while they were still using MyBPCare? If not, why?

If yes, how? Did you communicate with the patient’s GP about MyBPCare? If yes, how? Did you update the GP details on the patient’s dashboard? If not, what were the reasons?

1. Does the protocol for BP categorisation of community midwives often clash with yours?
2. What elements do we need to plan for in MyBPCare when a patient is being discharged back to primary care?
3. Do you have any questions?

**Demographics**

| Job role |  |
| --- | --- |
| Role in the study |  |
| Hospital/Community healthcare organisation |  |

**End of interview**: Thank them for participation.
