# Supplementary material for: Using qualitative research and the person-based approach to coproduce an inclusive intervention for postpartum blood pressure self-management
Source: BMJ Open. 2025 Jun 24;15(6):e098162. doi: 10.1136/bmjopen-2024-098162 (PMC12198848; doi:10.1136/bmjopen-2024-098162)
Supplement: online supplemental file 9 [file bmjopen-15-6-s009.docx]

**My BPCare intervention development Patient Interview topic guide**

**Qualitative Process study: Participant telephone interview schedule topic areas (intervention groups)**

- Remind the patient that the interview is to find out how you are getting on with MyBP Care ad that this will help improve the App, leaflet and how they all work.
- Check if they have understood the information sheet and completed the consent form.
- Ask them if they have any questions.
- Check if they are still happy to be interviewed and remind them that they can stop at any time if they wish to.
- Double check that they are ok for the interview to be recorded reminding them that the data will be anonymised. If yes, start recording.

**Introduction**

1. How are you doing? Can you tell me a little bit about your experience of blood pressure in this pregnancy? Probes: When were you diagnosed? When did you start taking the BP medication? Have you had to measure your own BP at home before? How did you record it? Did you use an App?

**Intervention**

1. I’m really interested in hearing about your experiences of using MyBP Care, can you tell me all about it? Probes: What were your thoughts when the midwife told you about it? How did you find the process of downloading the App? What were your first thoughts once you had the App? What did you think about the leaflet?
2. Could you tell me about anything that you have liked about My BP Care so far?
3. Can you tell me about things you disliked? Probes: Are there things you found confusing/hard to use/annoying?
4. Do you remember the first pages? What did you think of them?
5. How did you find the experience of measuring your BP and recording it into the App everyday? Probe: any challenges?
6. What do you think about the medication page? Have you tried to enter your own medicines? [If not could you have a look and] Please tell me what you think of it?
7. What did you think of the messages that came through the App? Probe: Any messages that were not clear or that we need to change?
8. What are your thoughts about the BP records page?
9. Have you had a chance to look into the more information pages? [If not, could you have a quick look and] Please could you let me know what you think?
10. If you forget to record your BP, a reminder message is sent to you, did you experience these? If yes, what did you think about them?
11. Can you tell me about anything else that you think might help improve the App and leaflet?
12. What are your thoughts on how to ensure people will use the App everyday?
13. Is there anything else you would like to tell me about MyBP Care that we haven’t already talked about?
14. Do you have any questions?

**Demographics**

| Age |  |
| --- | --- |
| Ethnicity |  |
| Highest Education qualification |  |
| Employment status |  |
| Post code |  |

**End of interview**: Thank them for participation. Ask their email address for the £20 love to shop voucher.
