# Supplementary material for: Using qualitative research and the person-based approach to coproduce an inclusive intervention for postpartum blood pressure self-management
Source: BMJ Open. 2025 Jun 24;15(6):e098162. doi: 10.1136/bmjopen-2024-098162 (PMC12198848; doi:10.1136/bmjopen-2024-098162)
Supplement: Supplementary file 11 [file bmjopen-2024-098162.reviewer_comments.pdf]

PEER REVIEW HISTORY

BMJ Open publishes all reviews undertaken for accepted manuscripts. Reviewers are asked to complete a checklist review form and are provided with free text boxes to elaborate on their assessment. These free text comments are reproduced below.

ARTICLE DETAILS

Title (Provisional)

Using qualitative research and the person-based approach to co-produce an inclusive intervention for post-partum blood pressure self-management

Authors

Ochieng, Cynthia Akelo; Burke, Carol; Green, Marcus; Igwe, Sandra; Tucker, Katherine Louise; Hinton, Lisa; Goddard, Lucy; Roman, Cristian; McManus, Richard J; Yardley, Lucy

---

VERSION 1 - REVIEW

**Reviewer**	**1**
**Name**	**Mei, Jenny Y**
**Affiliation**	**University of California Los Angeles**
**Date**	**08-Jan-2025**
**COI**	**None**

This is an intervention development study about a postpartum hypertension monitoring program being enacted by the NHS in England.

Overall the submission is quite long and I'm not sure all of the length is necessary. Specifically, I think there can be a shorter results section with less patient quotes.

It would be helpful to have more description on what protocols will be enacted as part of the program. For example, what are the blood pressure targets? What is the general workflow of how patients input blood pressures, who reviews the blood pressures, and who is in charge of making medication decisions? Do the patients go home with a blood pressure cuff? Are they all enrolled in the app and what happens if they don't have a smartphone? This will make the study more generalizable and useful outside of the study institution.

What is the goal of the program? This should be highlighted in the Methods section. Is the goal to reduce readmissions? Reduce maternal morbidity? Enhance patient satisfaction?

Given the authors brought up performing a RCT later, would recommend flushing out what the future goals and studies would be, and how they would be achieved.

Not sure overall purpose of publishing this. If it is like a descriptor of a RCT, would benefit from some preliminary data showing its likely usefulness.

**Reviewer**	**2**
**Name**	**Lewey, Jennifer**
**Affiliation**	**University of Pennsylvania**
**Date**	**18-Feb-2025**
**COI**	**None**

Title

1. Title: PPI is not defined in the title

Introduction

2. Clarification of the study objective is needed. The authors describe that prior postpartum BP self-management studies (SNAP-HT and POP-HT) have been shown to improve clinical outcomes. If that is the case, why is a new postpartum BP intervention needed?

3. To what degree are the authors adapting evidence-based interventions to a new context?

Methods

4. The Methods section may be easier to read if recruitment/participants across the 3 phases are described on one section, followed by a description of the 3 phases of intervention development.

5. Did the authors use an intervention development framework, such as Behavior Change Wheel or the MRC framework for complex interventions?

6. Given that 26 patients were recruited to participate in the pilot intervention, can the authors present any quantitative data to support the feasibility of the intervention, such as number of BP values reported, proportion who self-titrated medications, etc.

7. Given the interest in developing an intervention that can be implemented nationally, were there health system leaders or policy-makers invited to participate as stakeholder?

Results

Phase 1

8. The first paragraph describes the patient and clinician logic models. The description of the mediating processes in this section was difficult to follow. It would be helpful to discuss these processes in the context of the behavior change theory applied to intervention development. Presenting the logic model in the main manuscript may also help the reader follow the pathways to achieve the desired clinical outcomes.

Phase 2

9. I would recommend very briefly summarizing the results of the think-aloud interviews with patients and clinicians in this section. Consider moving the patient quotes to the supplement.

Phase 3

10. A table summarizing patient characteristics would be helpful. Please include more clinical details, such as whether all patients were discharged on BP medications or had a history of pre-pregnancy hypertension, as these clinical characteristics may impact their experience with the pilot intervention.

11. Similar to Phase 2, I would recommend more concisely summarizing the themes that emerged from interviews. In addition, this section may be easier to read if patient quotes were presented in a table which highlights the relevant themes.

12. Were there additional changes made to the intervention after analyzing the patient interviews in Phase 3?

13. The authors describe the process of developing the intervention, but it is not clear what the final intervention looks like. A summary of intervention components would be helpful, as well as visual demonstrating some of the components.

Discussion

14. Please comment on how the intervention developed using this approach differs from other published interventions designed to improve postpartum BP monitoring.

VERSION 1 - AUTHOR RESPONSE

Reviewer 1 feedback:

Overall the submission is quite long and I'm not sure all of the length is necessary. Specifically, I think there can be a shorter results section with less patient quotes.

Thank you, the entire manuscript and particularly the results section has been shortened and the interview quotes moved to supplementary file 5***.***

It would be helpful to have more description on what protocols will be enacted as part of the program. For example, what are the blood pressure targets? What is the general workflow of how patients input blood pressures, who reviews the blood pressures, and who is in charge of making medication decisions? Do the patients go home with a blood pressure cuff? Are they all enrolled in the app and what happens if they don't have a smartphone? This will make the study more generalizable and useful outside of the study institution.

Thank you, the protocol has been described more as:

Protocol

At the hospital, all recruited patients were set up on My BP Care app , provided with a free calibrated BP monitor and trained how to use both. There was the option of procuring smart phones for those who did not have a smart phone, however all patients recruited ended up having a smart phone.

Following discharge, patients checked and recorded their BP daily, receiving tailored feedback. Higher and lower values (in reference to NICE guidance) prompted them to contact their doctor within appropriate timelines. Simultaneously the patient's doctor would receive a message and flag via the intervention dashboard alerting them of the higher or lower readings requiring a medication review.

What is the goal of the program? This should be highlighted in the Methods section. Is the goal to reduce readmissions? Reduce maternal morbidity? Enhance patient satisfaction?

Thank you, we have added text in the methods section to highlight the goal:

The main purpose of this study was to develop an inclusive intervention for BP management post-partum that could be used among under-served communities and in different contexts. Success of the intervention will result in better BP management post-partum which is associated with reduced risk of developing hypertension and cardio-vascular disease.

Given the authors brought up performing a RCT later, would recommend flushing out what the future goals and studies would be, and how they would be achieved. Not sure overall purpose of publishing this. If it is like a descriptor of a RCT, would benefit from some preliminary data showing its likely usefulness.

We have included text in the discussion section clarifying the aim of this publication:

The aim of this publication is to document the process used to co-produce an inclusive intervention with underserved communities for blood pressure self-management. Blood pressure management post-partum is often haphazard and sub-par of the national recommendations. Underserved communities including black and ethnic minorities, those with lower income, education and living in more deprived areas are disproportionately affected and experience worse outcomes following HDPs. By co-producing an intervention with these populations and their clinicians, we were able to develop an intervention that is appropriate, effective, safe, and motivating-resulting in better management of BP post-partum. The resultant intervention is currently being trialed on a wider-scale to asses its impact on BP across the UK (https://www.isrctn.com/ISRCTN11042045).

Reviewer 2 feedback:

1. Title: PPI is not defined in the title
Thank you we, have now edited the title to describe the methodology applied more clearly: Using qualitative research and the person-based approach to co-produce an inclusive intervention for post-partum blood pressure self-management

Introduction
2. Clarification of the study objective is needed. The authors describe that prior postpartum BP self-management studies (SNAP-HT and POP-HT) have been shown to improve clinical outcomes. If that is the case, why is a new postpartum BP intervention needed?
We have now included some text clarifying that both SNAP-HT and POP-HT highlighted in their published findings that both studies lacked a diversity within their patients groups which needed to be incorporated into future studies, this study aimed to fill in those gaps by recruiting a diverse group of participants and patient and public involvement contributors. The updated text for clarification is:

Better management of HDPs post-partum is associated with reduced risk of developing chronic hypertension and longer-term cardio-vascular disease.[3]….

In previous studies (SNAP-HT and POP-HT), following self-management through a digital intervention for self-recording of BP, remote clinical oversight and subsequent medication adjustments, patients had better controlled blood pressure at six months post-partum and up to 4 years in longer term follow up.[8-10] However in both studies the patients were primarily white and middle class, findings from both studies recommend that further research be conducted with more diverse patient groups.[8, 10] This is particularly important given that prevailing disparities- maternal mortality is four times higher in black women than in white ones, 60% of deaths following pregnancy were associated with pre-existing conditions such as hypertension and ethnic minorities are over-represented among those deaths, multiple disadvantage like living in a more deprived area, being on lower income or lower education status further exacerbate worse outcomes.[11] Both POP-HT [10] and SNAP-HT [8] focused on patients with gestational hypertension, there was a need to develop an intervention that was suitable for patients from different ethnic backgrounds, living in more and less deprived areas, with different education levels and with HDPs including gestational and chronic hypertension. ~~Following the success in BP outcomes of the patients in SNAP-HT and POP-HT, there was a need to develop an intervention for self-monitoring that could be applied nationally through the NHS in the UK. This would need~~. The intervention needed to be useable not just in different NHS Trusts, primary and secondary care facilities but also by a diverse group of patients in different contexts.

3. To what degree are the authors adapting evidence-based interventions to a new context?

Learning from previous evidence-based interventions were utilized in this study as a basis for the co-production of the new intervention for this context suitable for a diverse range of patients with different socio-demographic characteristics, experiencing either chronic or gestational hypertension in different areas across the UK. Text added to state this:

Previous evidence-based interventions were utilised as a basis for the development of this intervention that would suit the diverse needs of the different patients and clinical contexts.[8, 9, 19-21]

Methods

4. The Methods section may be easier to read if recruitment/participants across the 3 phases are described on one section, followed by a description of the 3 phases of intervention development.

Thank you, we had previously attempted that approach but it resulted in more confusion with the reader not being able to distinguish which recruitment and procedures related to which phase. Given that the three phases were conducted chronologically, it follows that they are reported chronologically. We have not changed the manuscript but have included a sentence to clarify this in the methods section:

Each phase of development is explained below, in the order in which they were conducted.

5. Did the authors use an intervention development framework, such as Behavior Change Wheel or the MRC framework for complex interventions?

Yes, text is now included in the methods specifying this:
The MRC framework for complex interventions was applied in this study, it assists with prioritisation of research question, design and methods, and encourages the development and use of more promising approaches.[24] The person-based approach which is applied in this study was developed from the MRC guidance[25] as one of the promising approaches and is recognised within the MRC guidance as such.[26]

6. Given that 26 patients were recruited to participate in the pilot intervention, can the authors present any quantitative data to support the feasibility of the intervention, such as number of BP values reported, proportion who self-titrated medications, etc.
Thank you, the patients did not self-titrate their medication, through the intervention, they reported the medication they were on and updated that whenever their clinician advised a titration change. More detail has been added to give more details:
12 had chronic hypertension while 14 had gestational hypertension (including pre-eclampsia). They submitted a total of 499 BP readings with a median of 23 (minimum 3, maximum 43) and average of 20.8 (Standard Deviation 11.4). 53 medication changes were recorded of which 30 were self-reported by the patients and the rest were reported by their clinicians.

7. Given the interest in developing an intervention that can be implemented nationally, were there health system leaders or policy-makers invited to participate as stakeholder?
Yes, the study stakeholders included senior health system leaders and clinical government advisers. Text included to highlight this: (clinicians, researchers, senior health-system leaders, senior government advisers and third sector-based).

Results
Phase 1
***8. The first paragraph describes the patient and clinician logic models. The description of the mediating processes in this section was difficult to follow. It would be helpful to discuss these processes in the context of the behavior change theory applied to intervention development. Presenting the logic model in the main manuscript may also help the reader follow the pathways to achieve the desired clinical outcomes*.**

Thank you, text included to explain further:
In line with Bandura's social cognitive theory,[29] the proposed intervention needed to address the patients' personal (including cognitive) factors, behavioural factors and environmental (including social) factors. For example the intervention needed to impact the patient's knowledge, outcome expectation and attitude toward BP management. The intervention also needed to promote the patients' skills in BP self-management, and crucially promote their self-efficacy for BP self-management. The intervention also needed to incorporate environmental factors by promoting access to BP self-monitoring resources and provide a socially supportive environment through the clinical support for self-management. The intervention achieved these through containing…

Consistent with social cognitive theory, the mediating processes for this intervention involved cognitive factors such as…

Altering these cognitive factors impacted uptake and adherence to the intervention.

As with the patient logic model, the clinician logic model also aligned with social cognitive theory and represented the importance of targeting cognitive factors (outcome expectancies, attitudes and beliefs of BP self-monitoring through the intervention), behavioural factors (skills in supporting the intervention) and environmental factors (access to the intervention resources, and social norms within their individual clinical context).

Some of the barriers identified were: perceived the lack of time by mothers of newborns, mothers feeling too tired to self-manage their BP, forgetting to take their BP and medication, not feeling confident to take their own BP and engage with the intervention, feeling fine and not thinking that they need to take their BP or medication needing re-assurance of clinical oversight and medication safety. Barriers identified from the clinician perspective were: lacking capacity to support patients with the intervention, a lack of continuity of care into primary healthcare, uncertainty about the safety of self-management of BP post-partum. Facilitators were: the intervention aligning with current practice, possibility of more efficient management of patient medication titrations, and a chance to empower a diverse group of patients.

Phase 2

***9. I would recommend very briefly summarizing the results of the think-aloud interviews with patients and clinicians in this section. Consider moving the patient quotes to the supplement*.**

Thank you, quotes moved to the supplementary file 5 and text summarised as follows: Having designed and optimised the intervention based on the findings represented in the logic model and intervention planning table, subsequent participant think -alouds demonstrated that by adhering to the guiding principles (see table 1 above), the intervention was found to be feasible, appropriate, engaging and motivating…

Phase 3

***10. A table summarizing patient characteristics would be helpful. Please include more clinical details, such as whether all patients were discharged on BP medications or had a history of pre-pregnancy hypertension, as these clinical characteristics may impact their experience with the pilot intervention*.**

Thank you, a table with patient characteristics included as supplementary file 4. Clinical details also added in the text:

12 had chronic hypertension while 14 had gestational hypertension (including pre-eclampsia). They submitted a total of 499 BP readings with a median of 23 (minimum 3, maximum 43) and average of 20.8 (Standard Deviation 11.4). 53 medication plans were recorded of which 30 were self-reported by the patients and the rest were reported by their clinicians. All patients recruited were anticipated to be discharged on medication, however for some, their elevated BPs resolved quickly post-partum necessitating halting of medication.

***11. Similar to Phase 2, I would recommend more concisely summarizing the themes that emerged from interviews. In addition, this section may be easier to read if patient quotes were presented in a table which highlights the relevant themes*.**

Thank you, the section has been summarized and the quotes have been placed in a table attached as supplementary file 5. The summarized text is:
This user-testing was important as it highlighted whether the intervention was feasible, pragmatic, appropriate and motivating-the principles utilised to attain these are outlined in the guiding principles above. The interview findings demonstrated that the co-produced intervention had achieved these…

***12. Were there additional changes made to the intervention after analyzing the patient interviews in Phase 3*?**
Yes, text included to clarify this:
These were further utilised to optimise the intervention.

***13. The authors describe the process of developing the intervention, but it is not clear what the final intervention looks like. A summary of intervention components would be helpful, as well as visual demonstrating some of the components*.**
To enhance clarity, a section heading has been included labelled 'intervention'. This describes the intervention components. Also added is the sentence: Screenshots of the patient-facing elements of the digital intervention have been included as Supplementary file 10.

Discussion
***14. Please comment on how the intervention developed using this approach differs from other published interventions designed to improve postpartum BP monitoring*.**
Thank you, text added to illustrate this:
Interventions for the management of BP post-partum are an emerging area of research with a paucity of evidence of successful interventions.[6, 41] Published interventions include close clinical monitoring of patients [41], however this is often unattainable due to a lack of clinical capacity. Remote interventions for monitoring patients are being developed including using text messaging [42, 43] and other telehealth interventions. [44, 45] While these telehealth interventions are promising, they reported that they required a nurse to assess every patient's BP daily, make medication changes and avail themselves daily to all these patients. Due to the pragmatic constraints of that model within the UK NHS, our intervention ensured that alerts were sent to the clinical team regarding only the patients needing medication changes/closer monitoring freeing up clinical time that would have been spent on patients who do not need a change in their management. Other interventions have focused on education and other resources for lifestyle changes [41, 46] our study however identified that new mothers were not able or willing to make those lifestyle changes in the puerperium. This intervention has also build upon previous interventions developed in the UK [3, 8-10, 19, 20, 30] and optimised them to suit a diverse group of post-partum HDP patients being cared for in different clinical contexts.
